# Tuberculosis diagnosis and the complete drug resistance pattern from a single sample within a single day by use of a composite platform of MAX MDR-TB and AmPORE-TB

**DOI:** 10.1128/jcm.01388-25

**Published:** 2026-01-12

**Authors:** Harald Hoffmann, Andrey Golubov, Caroline Corbett, Dilfuza Allamuratova, Uladzimir Antonenka, Marion Heiß-Neumann, Sabine Hofmann-Thiel, Kripu Sharma, Laziz Turaev, Dzmitry Sinitski, Olim Kabirov

**Affiliations:** 1 Department IML red GmbH, WHO - Supranational Tuberculosis Reference Laboratory, Institute of Microbiology and Laboratory Medicine, Munich-Gauting, Bavaria, Germany; 2 SYNLAB Gauting, SYNLAB MVZ Dachau GmbH, Munich-Gauting, Germany; 3 Kuratorium Tuberkulose in der Welt e.V., Munich-Gauting, Bavaria, Germany; 4 Republican Specialized Scientific Practical Medical Centre of Phthisiology and Pulmonology under Ministry of Health of the Republic of Uzbekistan, Tashkent, Republic of Uzbekistan; 5 Asklepios Lungenklinik Gauting, Pulmonary Hospital, Munich-Gauting, Bavaria, Germany; 6 National Reference Laboratory, Republican Tuberculosis Hospital Machiton, Vahdat city, Shifo, Republic of Tajikistan; University of Western Australia, Perth, Australia

**Keywords:** tuberculosis, diagnostics, BD Max MDR-TB, AmPORE-TB, targeted next-generation sequencing, mWRD

## Abstract

Rapid TB diagnostics are essential for effective TB control. Combining WHO-recommended rapid molecular tests with downstream targeted next-generation sequencing (tNGS) enables faster drug resistance profiling. The objective of this study was to establish a one-day diagnostic platform (ODDP) integrating BD MAX MDR-TB and AmPORE-TB tNGS from a single sample. Pooled sputum samples spiked with 52 pre-characterized *Mycobacterium tuberculosis* (MTB) strains and 74 MTB-positive clinical samples were tested using BD MAX MDR-TB for TB, isoniazid, and rifampicin resistance. tNGS was performed from 5 µL of purified DNA leftover for each TB-positive sample in the BD MAX strips. IS6110/IS1081 Ct-values served as surrogate markers for TB DNA concentration. A total of 104 spiked and 60 clinical samples tested positive by BD MAX. The average time to the final ODDP result was 8.5 h. For samples with Ct ≤28, tNGS generated antibiotic resistance profiles for ≥12 antibiotics with 85.1% sensitivity in spiked and 73% in clinical samples. Failure rates were 10% and 8.3%, respectively. Resistance profiling most frequently (up to 11.3%) failed for clofazimine, pretomanid, and delamanid. The ODDP enables comprehensive TB diagnosis and resistance profiling from a single sample in 1 day. This platform can significantly accelerate the time to informed drug-resistant (DR)-TB treatment decisions.

**IMPORTANCE:**

Reducing the time to treatment initiation decreases patient drop-out rates, morbidity, the emergence of new drug resistances, and onward transmission of infection. Obtaining the complete resistome from the start is crucial for choosing a fully effective treatment regimen. Until now, diagnosis with full resistance profiling has required at least two sputum samples and 3 to 7 days for the complete workflow, obliging patients to return two to three times, which dramatically increased the risk of loss to follow-up. Our one-day diagnostic platform enables both diagnosis and comprehensive resistance testing from a single sample within 1 day. Patients can remain in a day clinic during testing and receive a fully effective, individualized treatment regimen the same day. This approach is expected to markedly reduce morbidity, drop-out rates, and transmission. The necessary instruments and technologies are already available in many high-prevalence countries and are currently being rapidly scaled up worldwide.

## INTRODUCTION

Tuberculosis remains a significant global health concern, with more than 10 million new cases occurring every year (1). An estimated 400,000 people developed drug-resistant (DR) TB in 2023, which poses a major threat to global TB control (2). Multidrug-resistant TB (MDR-TB) is defined by resistance toward isoniazid and rifampicin. Until 2022, MDR-TB was treated for 18–24 months with regimens consisting of up to seven different antibiotics with a cure rate ranging from 40% to 65% (2). Recently, the 9-month all oral, the 6-month BPaLM (bedaquiline, pretomanid, linezolid, moxifloxacin), and the 6-month BDLLfxC (bedaquiline, delamanid, linezolid, levofloxacin, clofazimine) regimens have not only substantially shortened treatment plans but also improved their outcomes (3–5). However, these regimens are only recommended when the susceptibility of the TB pathogen to the applied drugs has been confirmed (4, 6). Rapid diagnostics of drug susceptibility has always been essential for an improved treatment outcome (7, 8), but has become even more crucial since the advent of the highly effective, short anti-TB regimens.

The World Health Organization (WHO) has endorsed a large spectrum of molecular-biological, WHO-endorsed, rapid diagnostics tests (mWRD) that allow the diagnosis of TB and the detection of the most important genetic resistance markers for rifampicin and isoniazid to identify RR/MDR-TB with one or two tests (9). However, after having established the diagnosis of DR-TB, commercially available mWRDs only provide limited information on second-line anti-TB drugs, such as fluoroquinolones, thioamides, pyrazinamide, or the second-line injectable amikacin. Rapid tests on resistance markers for the drugs used in the BPaLM-, BDLLfxC-, and the 9-month all-oral regimens are still lacking. Targeted next-generation sequencing (tNGS) is not a rapid test *per se*; however, it can test for resistance toward all relevant anti-DR-TB drugs with high accuracy in a much shorter time than phenotypic testing of the bacteria (10). Therefore, many countries have started integrating tNGS into their diagnostic portfolios to more rapidly identify patients who are eligible for the shorter regimens (11). Three commercially available tNGS kits have been evaluated and partially endorsed by the WHO: the Deeplex MTB (Genoscreen, Lille, France), the AmPORE-TB (Oxford Nanopore Technologies [ONT], Oxford, UK), and the TBseq (Hangzhou ShengTing Medical Technology Co., Hangzhou, China). Besides their relatively high prices, their technical complexity still poses a major challenge to their integration into routine diagnostics (12). To use them most efficiently, they should be combined as downstream tests with cheaper and easier-to-use mWRD.

Combining tNGS with most of the available mWRDs requires a second sputum sample since the first sample is consumed by the initial tests. However, 10% to 35% of people with signs of TB drop out of follow-up between the collection of the first and second sputum samples (13–15). A solution to this challenge would be using the initial sputum sample for both mWRD and subsequent tNGS (16). The BD MAX MDR-TB (Becton-Dickinson, Frankley Lake, NJ) is a fully automated PCR-based method for detecting MDR-TB directly from sputum samples within a few hours. It provides 7 to 10 µL leftover DNA solution, which could be directly applied to tNGS to seek mutations associated with resistance toward second-line anti-TB drugs. Using this approach, a single sputum sample could be analyzed on an ultra-fast composite “one-day diagnostic platform” (ODDP) combining the BD Max MDR-TB test with subsequent tNGS using the Oxford Nanopore Technology, with results being available the same day. The objectives of this study were to (i) prove the feasibility of one-day TB diagnostics; (ii) validate the ODDP with spiked sputum samples; and (iii) confirm the diagnostic validity with clinical sputum samples.

## MATERIALS AND METHODS

### Study design

**Fig 1:**
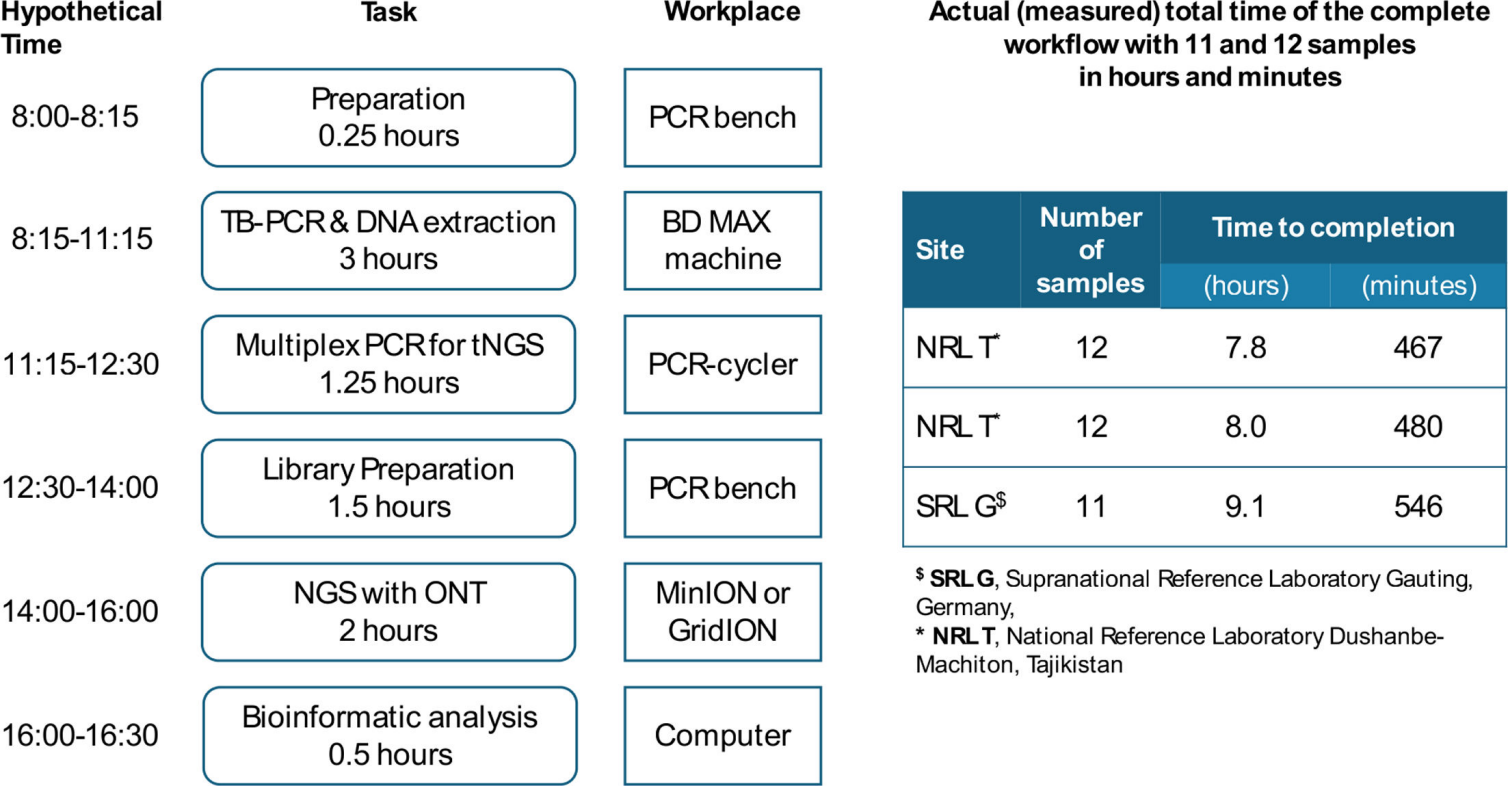
Hypothetical and measured times (in hours and minutes) of the one-day diagnostics platform combining the MAX MDR-TB (BD, NJ, USA) and the tNGS (ONT, Oxford, UK) tests, showing that the protocol can be completed within one working day.

Two centers, the WHO—Supranational TB Reference Laboratory Munich-Gauting, Germany, (hereinafter referred to as “SRL Gauting”), and the National TB Reference Laboratory in Machiton, Vahdat district, Tajikistan (hereinafter referred to as “TJK NRL”), applied spiked and clinical sputum samples without prior decontamination to the ODDP workflow that involved (i) sample inactivation with BD-STR reagent, (ii) starting the BD MAX MDR-TB test, (iii) collecting leftover DNA from the BD MAX extraction strips, (iv) running the AmPORE-TB tNGS PCR after receiving BD MAX results, (v) preparing the AmPORE tNGS library, (vi) MinION sequencing, and (vii) bioinformatic analysis and reporting of the results (Fig. 1). On each experimental day, eight to 22 samples plus one no-template and one positive control were tested.

In a first phase, the ODDP was validated with sputum samples spiked with pre-characterized *Mycobacterium tuberculosis* (MTB) strains at different concentrations. The number of aliquots and the concentrations per strain were selected based on our previous experience with the BD MAX system to ensure reliable and consistent Ct values (17). The characteristics of the spiked MTB strains are given in Table S1, and their test concentrations in Table S2 (both in the supplement). In a second phase, the diagnostic validity of the ODDP was evaluated with clinical samples (Table S1).

Spiked sputum samples were created using anonymized, naïve pooled leftover sputum from the diagnostic bacteriology department of the SYNLAB MVZ Augsburg (Germany), which was tested in 10 mL aliquots for the absence of TB by Xpert MTB/Rif ultra assay (Cepheid, USA), then pooled to 100 mL, thoroughly mixed, aliquoted to 5 mL, and stored at −20°C. A volume of 1.0 mL of this pool was spiked with 100 µL suspension of pure MTB strains to a final bacterial concentration according to Beutler et al. (17).

Clinical samples were collected from people with TB and immediately anonymized at the Asklepios Pulmonary Hospital, Gauting (Bavaria, Germany), and the Republican TB Hospital of Machiton (Vahdat District, Tajikistan).

### BD MAX

The BD MAX MDR-TB test (BD, USA) was performed following the manufacturer’s instructions and as previously described (18, 19). The Ct values of the IS6110/IS1081 target were recorded together with the results reported by the BD MAX for TB positivity, isoniazid, and rifampicin resistance. A volume of 5 µL of the purified DNA solution leftover in each BD MAX MDR-TB extraction strip was taken for tNGS on the same day.

### Targeted and whole-genome next-generation sequencing

The AmPORE-TB tNGS assay targets 25 regions of 24 resistance-associated genes (plus *hsp65* for NTM identification and the spoligotyping region) and provides antibiotic resistance profiles (ARPs) covering up to 16 antibiotics (Table S3). Valid target results are those where either a mutation was identified or no mutation was found, but a valid control was present (indeterminate results or those with failed internal controls were considered invalid). Multiplex-PCRs and subsequent libraries were prepared from 5 µL BD MAX purified DNA with the Invitrogen Platinum II Taq Hot-Start DNA Polymerase using the primers and reagents coming with the AmPORE-TB kits according to the manufacturer’s instructions (20). For each set of eight to 22 samples, an internal control, a positive control (both provided by the manufacturer), and a no-template control (PCR-grade water) were included. tNGS libraries were quantified using the Qubit 1X dsDNA High Sensitivity (HS) Kit on a Qubit 3.0 Fluorometer. Following the attachment of the Rapid Adapter F, tNGS libraries were loaded into the FLO-MIN106D cartridge and sequenced on a MinION (MIN-101B) device (ONT, Oxford, UK). Depending on the number of samples, sequencing runs lasted 45–90 min to reach ~1 Mio reads. Flow cells were washed using the Flow Cell Wash Kit (EXP-WSH004, ONT, UK) after each run and used for a total of four runs. Initially, data analysis was performed using the EPI2ME Labs V4.1.0 software and the wf-tb-amr-v2.0.0-alpha1 pre-release version of the *M. tuberculosis* workflow (ONT, UK). Later, the data were re-analyzed using the newer version of the EPI2ME Labs V5.1.9 software and the workflow v2.0.0-beta2.

Whole-genome sequencing (WGS) was performed from cultured bacteria. WGS libraries were prepared using the Nextera XT Library Preparation and Index Kits (Illumina, San Diego, CA, USA) following the manufacturer’s instructions as previously described (12), and sequenced on a MiniSeq sequencer using a High Output Cartridge in a paired-end run (2 × 151 bp). After demultiplexing, fastq files were analyzed using the online PhyReSe pipeline (21). Mutations identified were interpreted according to the latest WHO recommendations in the second catalog of resistance-associated mutations (22).

### Statistical analysis

Samples were categorized into the groups of 16 antibiotics (ABs), ≥14 ABs, ≥12 ABs, and ≥10 ABs based on the minimum number of ABs covered by the ARPs generated by tNGS. Statistical analyses were performed using Stata 16.0 (StataCorp LP, College Station, TX). The limit of detection (LoD_95_), i.e., the minimum bacterial concentration in the sputum matrix that yielded positive results in 95% of test runs, was calculated using GraphPad Prism 8.0.2 (GraphPad Software, USA) as previously described (17). Comparisons between the number of antibiotics detected (for all categories) were analyzed for significance using multivariate tests of significance, Student’s *t*-test, and the Kruskal-Wallis test, with a *P* value of ≤0.05 being considered significant.

## RESULTS

### Timeline and workflow

To evaluate the feasibility of TB diagnostics within 1 day from the bacteriological confirmation by the MAX MDR-TB assay to the determination of the full resistome by tNGS, the ODDP was tested in three independent runs with 11 to 12 samples each at both study sites, the NRL of the low-income, high TB prevalence and high DR-TB burden country, Tajikistan, and the SRL Gauting in the high-income, low-TB burden country, Germany. On average, the complete diagnostic workflow was completed within an 8.5-h timeframe (Fig. 1), corresponding to a workday, including a 30 min lunch break.

**Fig 2:**
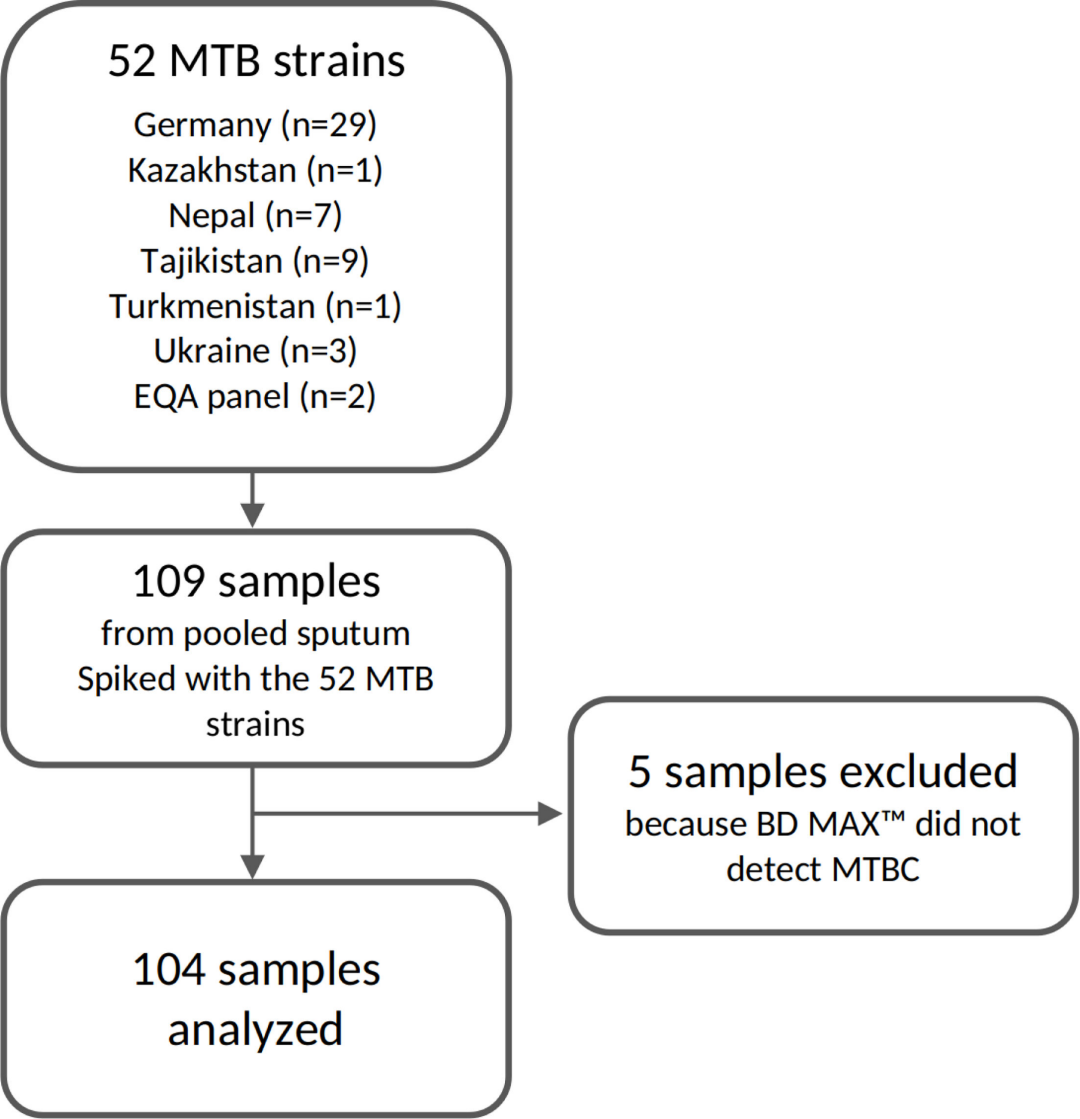
Samples included in the analyses tested in SRL Gauting.

**Fig 3:**
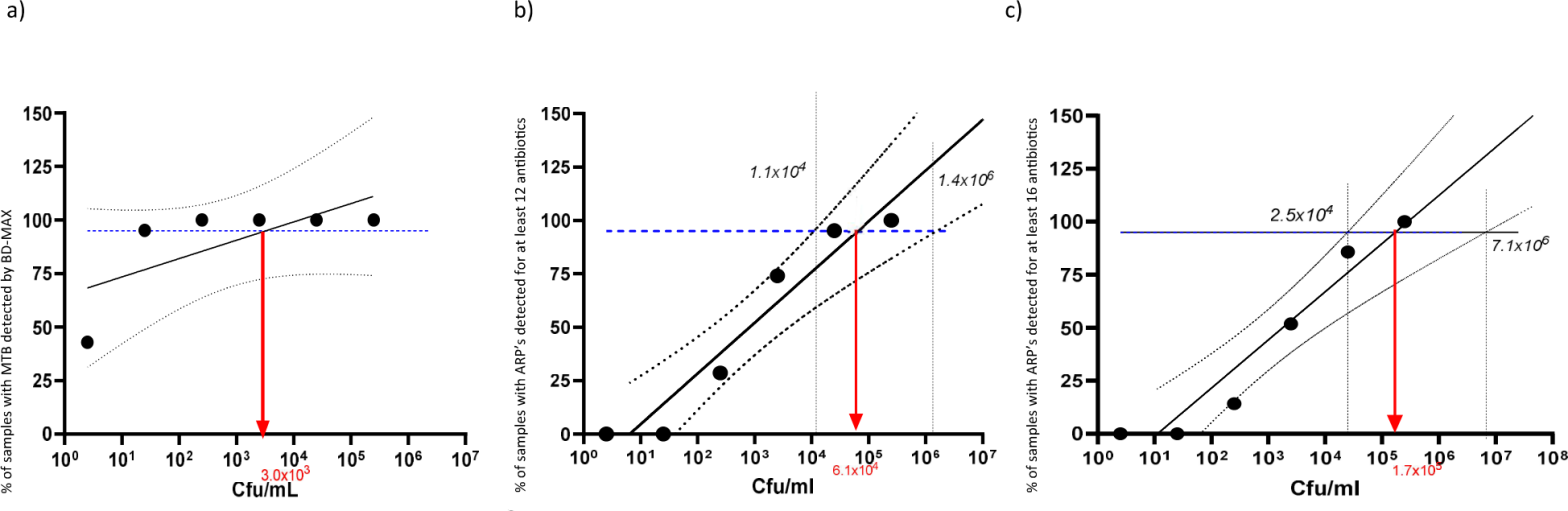
LoD_95_ for (**a**) detecting the *M. tuberculosis* marker IS6110/1081 with the BD MAX; (**b**) producing valid antibiotic resistance patterns for ≥12 AB by tNGS with the DNA isolated by BD MAX; and (**c**) detecting ARP’s for 16 antibiotics using tNGS with the DNA isolated by BD MAX. All LoDs were determined with spiked samples. The red arrow indicates the calculated LoD_95_. The solid black line is the predicted fit of samples; blue dotted line: 95% of samples meeting the respective criteria detected; black dashed lines: upper and lower 95% confidence intervals. The x-axis shows the log10 of the cfu/mL.

**Fig 4:**
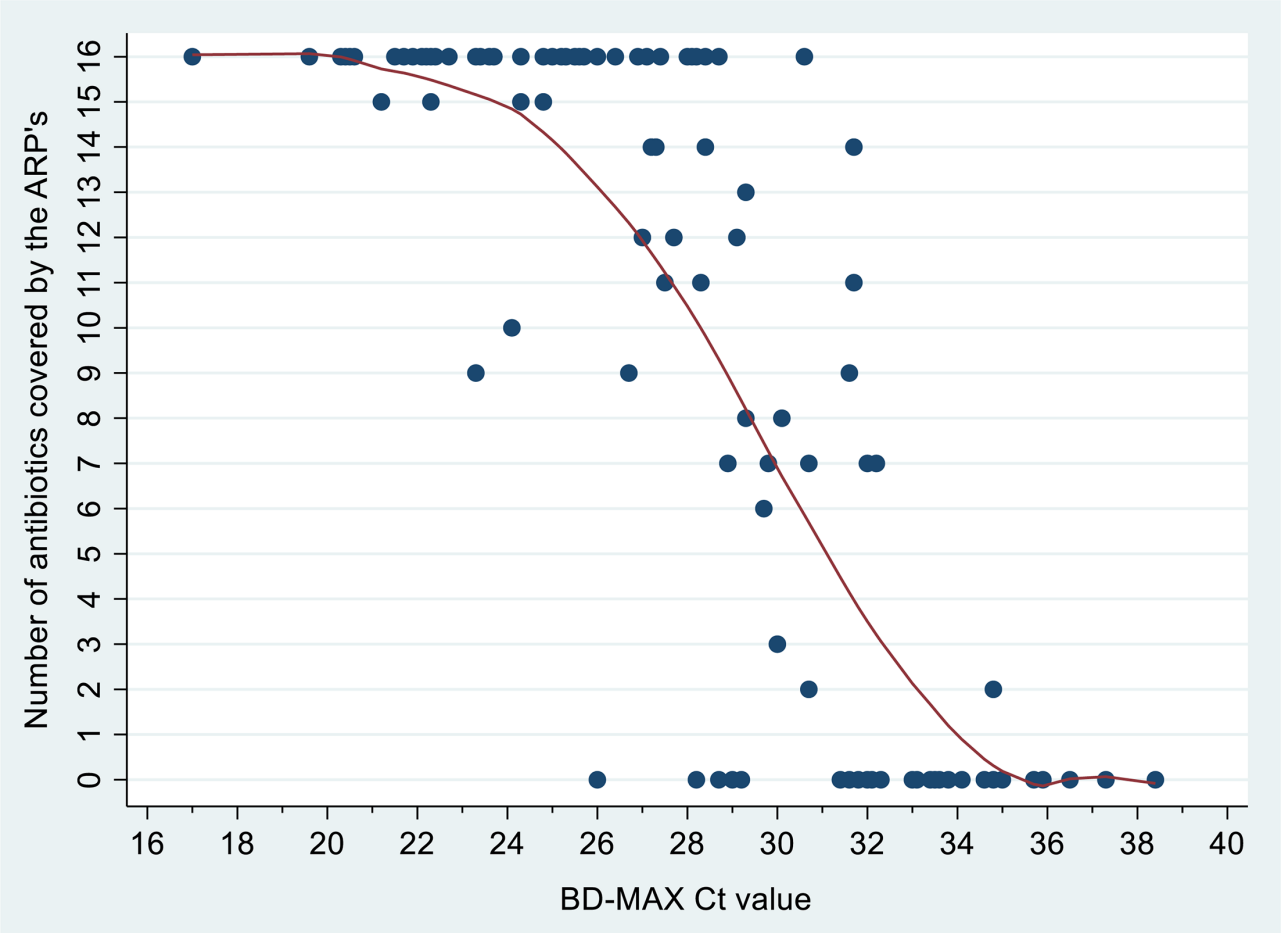
The number of antibiotics that tNGS reported with the ARPs for the 104 spiked samples and the corresponding BD MAX Ct values for the IS6110/IS1081 target in the MAX MDR-TB assay.

For the technical validation of the ODDP, 109 aliquots of pooled sputum samples were spiked with TB bacteria (Fig. 2; Table S2). The LoD_95_ of the BD MAX MDR-TB test was determined to be 3.0 × 10^3^ cfu/mL (Fig. 3a). BD MAX MDR-TB failed to detect MTBC in five spiked samples (4 × 10^0^, 1 × 10^1^ cfu/mL); they were excluded from downstream tNGS analysis. The mean Ct value of the IS6110/IS1081 target included in the BD MAX MDR-TB test was 28.2 (range = 17.0–38.4; Table 1). Ct values of the single copy *devR* gene in the BD-MAX 530/565 channel were an average of 3.6 (range: 1.4–5.4) higher than IS6110/IS1081. Of the 104 samples with positive BD-MAX results, 19% (*n* = 20) of the *devR* results fell into the non-linear range above Ct 35, with an additional 10% (*n* = 10 samples) not yielding *devR* PCR products at all. While Ct values of samples with ARPs covering 16 antibiotics were clearly distinguishable from samples with complete tNGS failure (ARP covering 0 antibiotics), only a trend for decreasing Ct values with increasing numbers of antibiotics covered by the tNGS ARPs was identified (Fig. 4).

**TABLE 1:** BD MAX Ct values for spiked sputum samples grouped by the tNGS ARPs covering the indicated number of ABs

AB covered by ARP	Number of observations with ARP detection	Mean Ct value	Standard deviation (Ct value)	Min Ct value	Max Ct value	*P* value^*a*^
16	41 (39.4%)	24.3	2.9	17.0	30.6	<0.001
<16	63 (60.6%)	30.7	3.7	21.2	38.4	
≥14	49 (47.1%)	24.5	3.0	17.0	31.7	<0.001
<14	55 (53.9%)	31.4	3.2	23.3	38.4	
≥12	53 (51.0%)	24.8	3.1	17.0	31.7	<0.001
<12	51 (49.0%)	31.6	3.2	23.3	38.4	
≥10	57 (54.8%)	25.0	3.2	17.0	31.7	<0.001
<10	47 (45.2%)	31.9	3.0	23.3	38.4	
≥0	104 (100%)	28.2	4.6	17.0	38.4	

^*a*^ Student’s *t*-test for the mean Ct values.

### Spiked sputum BD MAX and tNGS results

Using purified leftover DNA generated by BD MAX from 104 spiked sputum samples that tested positive for MTBC DNA via the BD MAX MDR-TB assay, the LoD_95_ of tNGS was determined to be 6.1 × 10⁴ CFU/mL (Fig. 3b; 95% CI: 1.1 × 10⁴–1.4 × 10⁶ CFU/mL) for successful sequencing of resistance genes to ≥12 antibiotics, and 1.7 × 10⁵ CFU/mL (Fig. 3c; 95% CI: 2.5 × 10⁴–1.7 × 10⁶ CFU/mL) for ≥16 antibiotics. tNGS successfully sequenced resistance genes for ≥12 antibiotics with 53/104 samples (51.0%; mean IS6110/IS1081 Ct value: 24.8), and for 16 antibiotics with 41/104 samples (39.4%; mean Ct value: 24.3), respectively (Table 1). Mean Ct values were consistently lower (*P* < 0.001) in samples where tNGS validly sequenced resistance genes for ≥10, ≥12, ≥14, or 16 antibiotics, compared to those where this was not achieved.

**Fig 5:**
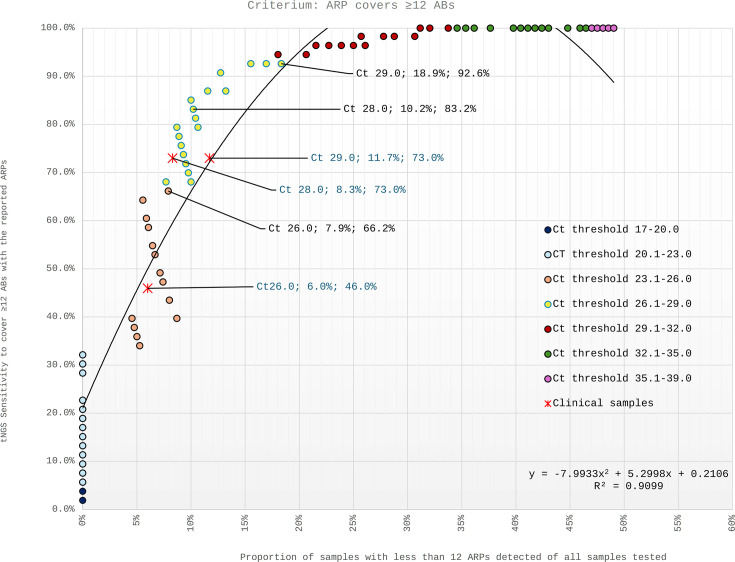
BD MAX Ct values for the IS6110/IS1081 target and ARPs covering ≥12 ABs. Each dot represents the Ct value of at least one spiked sample, with color indicating the corresponding Ct value range. The y-axis shows the proportion of samples with Ct values equal to or lower than the indicated value that yielded ARPs covering ≥12 antibiotics, relative to all spiked study samples with ARPs ≥12 antibiotics, irrespective of their Ct values. The x-axis shows the proportion of tests that failed to produce ARPs covering ≥12 antibiotics among all samples tested (*n* = 104). Red asterisks (Ж) denote the BD MAX Ct value thresholds of 26, 28, and 29 used for the 60 clinical samples. The percentages displayed for the highlighted dots and asterisks correspond to their respective x- and y-axis values.

This finding suggests that BD MAX Ct values could serve as predictors for the likelihood of successful downstream tNGS diagnostics. In Fig. 5, all IS6110/IS1081 Ct values measured from spiked samples are represented as individual dots with color codes each corresponding to a specific Ct range (e.g., yellow for Ct 26.1–29.0). The y-axis represents the technical sensitivity of tNGS when using the indicated Ct value as a cutoff for downstream tNGS testing. It shows the proportion of samples at or below the given Ct value, with ARPs covering ≥12 antibiotics out of all spiked study samples with ARPs covering ≥12 antibiotics (disregard the Ct value). The x-axis shows the probability that tNGS produces ARPs covering less than 12 antibiotics, i.e., the proportion of samples at or below the given Ct value that had ARPs <12 antibiotics out of all samples tested in the study.

Example: 39 (37.5%) of the 104 spiked samples used for this blot had an IS6110/IS1081 Ct value higher than, and 65 (62.5%) equal to or lower than 28. If choosing a Ct threshold of 28, the technical sensitivity would be 83.2% and the risk of tNGS failing to cover 12 antibiotics would be 10.2%. In practical terms, if 100 samples theoretically generate 67 ARPs ≥12, applying a Ct ≤28 cutoff would test 62 samples and return 56 successful ARPs ≥12 and six with <12. The same logic applies to ARPs covering all 16 antibiotics (Fig. S1).

### Clinical samples

A total of 74 clinical sputum samples (7 from Germany, 67 from Tajikistan, Table S1) were subjected to the ODDP. The BD MAX MDR-TB qPCR was negative with 10 (13.5%), and failed with 4 (5.4%; 2× inhibition, 2× technical error) of them. For the remaining 60 clinical samples, the mean IS6110/IS1081 Ct value was 28.3 (range: 18.3–39.5). tNGS produced ARPs covering ≥12 and 16 antibiotics with 37/60 (61.7%) and 27/60 (45.0%) clinical samples, respectively (Table 2). The sensitivity rates to determine ARPs covering ≥12 antibiotics were 45.9% and 73.0% for the Ct value thresholds of 26 and 28, respectively (Fig. 5). For the same thresholds, the proportions of “unsuccessful” tNGS (definition see above) were 6.0% and 8.3%, respectively.

**TABLE 2:** The number of clinical samples with IS6110/IS1081 Ct values within the indicated ranges for the ARPs covering 16 and ≥12 antibiotics and the corresponding other samples for which the ARPs covered less than 16 and 12 ABs, respectively

ARP coverage	Number of samples with IS6110/IS1081 Ct value			Total
	<26.0	26.1–28.0	>28.1	
16 ABs	13 (48%)	7 (26%)	7 (26%)	27 (100%)
≤15 ABs	8 (24%)	4 (12%)	21 (64%)	33 (100%)
≥12 ABs	17 (46%)	10 (27%)	10 (27%)	37 (100%)
≤11 ABs	4 (18%)	1 (4%)	18 (78%)	23 (100%)

### Antibiotics falling out of the tNGS antibiotic resistance profiles

Among the samples with ARPs covering ≥12 antibiotics, clofazimine, pretomanid, and delamanid failed in 5.7% to 11.3% of tests and were not covered in ≥20% of ARPs reporting at least one valid antibiotic with both, spiked (*n* = 71) and clinical (*n* = 46) samples (Table 3). Ethambutol dropped out of the ARPs in >20% with clinical, and streptomycin, capreomycin, and ethionamide in >20% with spiked samples. In contrast, bedaquiline did not fail in any spiked samples and failed in only one clinical sample (2.7%).

**TABLE 3:** Number of ABs for which tNGS did not produce valid results due to insufficient sequencing quality stratified for the ARPs that covered 16, ≥14, *≥*12, *≥*10*, and ≥*1 *antibiotics^a^*

Antibiotic	ABs not included in the ARPs covering 16 ABs		ABs not included in the ARPs covering ≥14 ABs		ABs not included in the ARPs covering ≥12 ABs		ABs not included in the ARPs covering ≥10 ABs		ABs not covered in ARPs covering ≥1 AB	
	Spiked sputum (*n* = 41)	Clinical samples (*n* = 27)	Spiked sputum (*n* = 49)	Clinical samples (*n* = 33)	Spiked sputum (*n* = 53)	Clinical samples (*n* = 37)	Spiked sputum (*n* = 57)	Clinical samples (*n* = 38)	Spiked sputum (*n* = 71)	Clinical samples (*n* = 46)
Isoniazid	0	0	0	0	0	0	0	0	3	3
Rifampicin	0	0	0	0	2	0	3	0	11	3
Ethambutol	0	0	0	2	0	3	0	3	7	**11**
Pyrazinamide	0	0	0	1	1	1	2	2	6	7
Levofloxacin	0	0	1	0	1	1	2	1	9	3
Moxifloxacin	0	0	1	0	1	1	2	1	9	3
Bedaquiline	0	0	0	1	0	1	0	2	5	7
Linezolid	0	0	1	0	1	0	1	1	8	3
Clofazimine	0	0	1	1	3	3	5	4	**17**	**12**
Pretomanid	0	0	2	1	6	4	9	5	**23**	**12**
Delamanid	0	0	2	1	6	4	9	4	**23**	**11**
Streptomycin	0	0	2	1	2	1	6	1	**16**	7
Amikacin	0	0	0	0	0	0	0	0	6	4
Capreomycin	0	0	3	1	4	2	8	2	**20**	9
Kanamycin	0	0	0	0	0	2	1	3	7	9
Ethionamide	0	0	2	0	3	0	3	0	**14**	3

^*a*^ Spiked and clinical sputum samples are separated for each ARP category. The numbers exceeding 20% of each column are highlighted in bold letters.

### Quality assurance

BD MAX MDR-TB agreed with tNGS in 35/41 (85.4%), 66/66 (100%), and 41/47 (87.2%) of valid *inhA*, *katG*, and *rpoB* results, respectively (Table 4). The repeatability of the ODDP was evaluated with sputum samples spiked with three strains in different concentrations, two tested twice, one tested three times, partly by different technicians on different days (Table S4). At a bacterial concentration of 10^4^ cfu/mL (mean BD MAX Ct value 23.1; range: 20.3–30.6; SD: 3.41), tNGS reported valid results for all 16 antibiotics with all repeats. With lower bacterial concentrations, the number of antibiotics covered by tNGS dropped gradually to zero with 6/7 samples at a concentration of 10 cfu/mL. In samples spiked with strains IML-00180 or IML-00771, tNGS misclassified the *rpoB* or *embB* genes as wild types, although they had mutations that were both confirmed by WGS. The repeatability dropped with declining bacterial concentrations.

**TABLE 4:** Number of samples with ARPs covering isoniazid (INH; katG and inhA) and rifampicin (RIF; rpoB) for 104 spiked samples using tNGS and BD MAX^*b*^^,^^*c*^

		BD MAX			
Gene	tNGS	Mutation	Wild type	IND/UNR	Total
inhA					
	Mutation	**11**	5^*a*^	1	17
	Wild type	1	**24**	1	26
	ND/UNR	3	42	**16**	61
	Total	15	71	18	104
katG					
	Mutation	**54**	0	0	54
	Wild type	0	**12**	1	13
	ND/UNR	18	3	**16**	37
	Total	72	15	17	104
rpoB					
	Mutation	**32**	0	9	41
	Wild type	6	**9**	4	19
	ND/UNR	15	4	**25**	44
	Total	53	13	38	104

^*a*^ Four samples with inhA_c.-154G>A and one sample with inhA_p.Ser94Ala.

^*b*^ ND, not detected, UNR, unreportable (e.g., due to inhibition, technical reasons).

^*c*^ The bold letters reflect the matching cases with equal outcomes of both BD MAX and tNGS.

Twenty samples spiked with different strains that underwent the ODDP were re-analyzed by WGS. Three hundred wild-type genes and 111 mutations reported by tNGS were reproduced by WGS. In 13 strains, tNGS reported 20 genes as wild type, whereas WGS identified mutations in the same targets (one each in *inhA*, *pncA*, *rplC*, two in *ethA*, four in *rrs*, five in *embB*, and six in *rpoB*; Table 5). tNGS identified mutation *eis*_n.1401A>G, where WGS reported wild type. Seven, five, and two strains showed one, two, and three, respectively, discrepancies between tNGS and WGS. The number of invalid results for the 25 tNGS targets correlated with the number of discrepancies between tNGS and WGS (Fig. S1; Poisson regression model; *P* = 0.013). Each additional valid tNGS target reduced the risk of a discrepancy by 11% (incidence rate ratio = 0.89; 95% CI 0.80, 0.97).

**TABLE 5:** Discrepancies in mutations identified between tNGS and WGS^*a*^

Sample ID	cfu	is.ct	Valid tNGS targets	Target gene	BD MAX	tNGS	WGS-Profiler	WGS-PhyResSe
IML-02051	10^3^	27,7	17	*eis*	na	n.1401A>G	wt	wt
IML-00757	10^3^	24,1	17	*embB*	na	wt	p.Asp1024Asn	p.Asp1024Asn, p.Leu172Arg
IML-01709	10^3^	26,7	16	*embB*	na	wt	p.Met306Ile	p.Met306Ile
IML-02051	10^3^	27,7	17	*embB*	na	wt	p.Met306Ile	p.Met306Ile
IML-02285	10^2^	27,2	23	*embB*	na	wt	p.Met306Val	p.Met306Val
IML-03823	10^5^	21,5	20	*embB*	na	wt	p.Tyr334His	p.Tyr334His
IML-00755	10^2^	29,1	18	*ethA*	na	wt	c.1427_1428dupAG	ins C - CCT
IML-00765	10^3^	25	25	*ethA*	na	wt	c.1427_1428dupAG	ins C - CCT
IML-00768	10^3^	23,3	14	*inhA*	mut	wt	c.-777C>T	c.-777C>T
IML-02052	10^2^	30,7	15	*pncA*	na	wt	p.Gly132Ser	p.Gly132Ser
IML-02052	10^2^	30,7	15	*rplC*	na	wt	p.Cys154Arg	p.Cys154Arg
IML-00655	10^4^	22,3	25	*rpoB*	i	wt	p.Ser450Leu	p.Ser450Leu
IML-00755	10^2^	29,1	18	*rpoB*	i	wt	p.Ser450Leu	p.Ser450Leu
IML-00757	10^3^	24,1	17	*rpoB*	mut	wt	p.Gln429Leu	p.Gln429Leu
IML-00757	10^3^	24,1	17	*rpoB*	na	wt	p.His445Tyr	p.His445Tyr
IML-00768	10^3^	23,3	14	*rpoB*	mut	wt	p.His445Leu	p.His445Leu
IML-02307	10^3^	24,8	23	*rpoB*	mut	wt	p.Ser450Leu	p.Ser450Leu
IML-00155	10^3^	24,8	26	*rrs*	na	wt	n.517C>T	n.517C>T
IML-00768	10^3^	23,3	14	*rrs*	na	wt	n.514A>C	n.514A>C
IML-00771	10^4^	20,4	25	*rrs*	na	wt	n.514A>C	n.514A>C
IML-01709	10^3^	26,7	16	*rrs*	na	wt	n.1401A>G	n.1401A>G

^*a*^ na = not analyzed; i = indeterminate; mut = mutation; wt = wildtype.

## DISCUSSION

The tremendous potential of net-generation sequencing-based prediction of *M. tuberculosis* drug resistance has been amply proven (23, 24). Combining the BD MAX system with tNGS on an ONT platform is a promising paradigm for ultra-fast TB diagnostics, including comprehensive resistance profiling of MDR-TB and XDR-TB, within a single workday from a single sputum sample. This approach integrates the speed and precision of a WHO-recommended rapid diagnostic test for TB diagnostics with the depth of NGS technologies for resistance testing, offering a potentially transformative solution for both clinical and public health applications. In everyday clinical outpatient management, patients are frequently lost when recalled to provide a second sputum sample (13, 14). Combining the BD MAX MDR-TB with the AmPORE-TB assay, all analyses are performed from a single sample so that the patient does not need to return until the diagnosis is fully established and an informed treatment decision has been made.

While the time to result of culture-based, phenotypic diagnostics takes 4 to 6 weeks (25) and of WGS from positive cultures 2 to 4 weeks, several previous studies investigated the potential of its reduction to 3 to 8 days on average by combining an initial mWRD with a downstream tNGS (10, 16, 26). Our ultra-fast ODDP is capable of reducing this time even further to a single day, provided that the DNA of the MTBC is sufficiently concentrated in the sample. The BD MAX MDR-TB assay offers this opportunity by providing leftover purified DNA after test completion for downstream tNGS analysis, while most other mWRDs consume the complete DNA for their tests. People seeking medical diagnosis at a TB dispensary could thus theoretically wait for the complete results before returning back home, which will most likely substantially reduce the patient drop-out rate.

Using the Ct value of the IS6110/IS1081 target, the BD MAX MDR-TB assay provides a powerful measure to discriminate samples for which tNGS will most likely be successful from those for which tNGS will most likely fail. When using the Ct of 28.0 as an orientation mark, tNGS determines ARPs covering at least 12 antibiotics with 73% of clinical samples for which this is technically possible. At the same time, less than 10% of the ARPs cover less than 12 antibiotics with all samples that will be tested. Such a resource-saving approach is of particular importance for laboratories in low- to middle-income countries where more than 90% of the worldwide TB cases occur.

Noteworthy, 73% of the antibiotic resistance patterns covering at least 12 antibiotics in fact covered all 16 antibiotics included in the assay. However, a pattern of 12 antibiotics seems to be a reasonable quality target since key antibiotics like bedaquiline, linezolid, and moxifloxacin were included in 97% to 100% of the ARPs covering ≥12 antibiotics. Less fortunate is the fact that pretomanid, a key drug of the 6-month BPaLM regimen, failed in up to 11% of tNGS runs covering ≥12 antibiotics. The AmPORE-TB assay determines pretomanid resistance by analyzing the genes *ddn*, *fbiA*, *fbiB*, *fbiC,* and *fgd1*. Other reported tNGS assays, including the most widespread Deeplex Myc-TB (Genoscreen, France), do not include pretomanid at all (10, 26–29). Furthermore, the knowledge of genetic mutations associated with pretomanid resistance is still scarce; however, this gap might close soon with the arrival of new updates in the third edition of the WHO mutation catalog (30).

The potential of tNGS with regard to resistance testing of TB bacteria has been proven by other studies (31, 32). A recent comparison of AmPORE-TB with the Deeplex Myc-TB assay showed 100% concordance with regard to the detection of mutations covered by both assays (24). Correspondingly, Colman et al. reported similar diagnostic sensitivity and specificity rates of AmPORE-TB and Deeplex Myc-TB for the majority of drugs, although they likely used one of the earlier versions of the ONT bioinformatic pipeline that was based on the first WHO mutation catalog (32, 33).

We repeated our analyses using the latest ONT software based on the most comprehensive mutation catalog available, which performs markedly better than the previous alpha version. Despite the overall high quality of the AmPORE-TB assay, we identified some limitations in its repeatability. When the assay failed to validly sequence ≥1 targets, the likelihood of misclassifying mutations as wild type increased, particularly for the *rpoB*, *embB*, and *rrs* genes. Such sequencing failures indicate low concentration or suboptimal quality of target DNA, which poses a still not fully understood technical risk of incorrect sequencing or misinterpretation during bioinformatic analysis (34).

According to the manufacturer’s validity criteria, a result is considered reliable when at least 10 resistance-associated genes are covered (20), whereas a recent AmPORE-TB field evaluation proposed a higher threshold of ≥15 genes (35). Integrating that evidence with our findings suggests the following decision-making algorithm: if BD MAX yields an IS6110/IS1081 Ct value ≥28, downstream tNGS is recommended, as it is likely to provide resistance information for at least 12 antibiotics with minimal risk of assay failure. When the tNGS profile covers fewer than 15 antibiotics (assuming compliance with internal quality control, i.e., ≥20× coverage of both *hsp65* and the internal process control), laboratories may regard detected resistance-associated mutations as reliable and report them to the clinician, but should withhold wild-type results until sequencing is repeated from cultured material or phenotypic drug susceptibility testing is completed. Discrepancies might be resolved by sequencing whole genes from cultured bacteria.

Besides the strengths of the extensive validation of our proposed ODDP regarding repeatability, reproducibility, and congruence with alternative methods like WGS, our study certainly also has its weaknesses. The total time to result was only measured in two laboratories on three different days and should be further determined in more extensive multi-center studies. The number of clinical samples was still limited and should also be enlarged in future studies initiated in different areas of the world to reach the WHO prequalification criteria (36). The genotypes were limited to only two major MTB lineages. Future studies should include more MTB bacteria, particularly from lineages 1, 3, 5, 6, and 7.

In summary, our study presents the ODDP as a robust diagnostic platform for TB from bacteriological confirmation of the disease to the provision of a complete drug resistance pattern covering at least 12 antibiotics from a single sample within a single day. Future clinical studies might elucidate the extent to which such ultra-fast diagnostics improve clinical management of people with TB and their treatment outcomes.

## SUPPLEMENTAL MATERIAL

Supplemental tables and figures jcm.01388-25-s0001.pdf Tables S1 to S4, and Figures S1 and S2.

ASM does not own the copyrights to Supplemental Material that may be linked to, or accessed through, an article. The authors have granted ASM a non-exclusive, world-wide license to publish the Supplemental Material files. Please contact the corresponding author directly for reuse.
