# Supplementary material for: Tuberculosis diagnosis and the complete drug resistance pattern from a single sample within a single day by use of a composite platform of MAX MDR-TB and AmPORE-TB
Source: J Clin Microbiol. 2026 Jan 12;64(2):e01388-25. doi: 10.1128/jcm.01388-25 (PMC12892996; doi:10.1128/jcm.01388-25)
Supplement: Supplemental tables and figures — Tables S1 to S4, and Figures S1 and S2. [file jcm.01388-25-s0001.pdf]

Tuberculosis diagnosis and the complete drug resistance pattern from a single sample within a single day by use of a composite platform of MAX™ MDR-TB and AmPORE-TB

Harald Hoffmann[1,2,3§], Andrey Golubov[1§], Caroline Corbett[1§], Dilfuza Allamuratova[4], Uladzimir Antonenka[1,3], Marion Heiß-Neumann[3,5], Sabine Hofmann-Thiel[1,2], Kripu Sharma[1,2], Laziz Turaev[4], Dzmitry Sinitski[1$], Olim Kabirov[6$]

[§]equally contributing first authors [$]equally contributing last authors

Affiliations:

1. Institute of Microbiology and Laboratory Medicine, Department IML red GmbH, WHO - Supranational Tuberculosis Reference Laboratory, Munich-Gauting, Bavaria, Germany
2. SYNLAB Gauting, SYNLAB MVZ Dachau GmbH, Munich-Gauting, Germany
3. Kuratorium Tuberkulose in der Welt e.V., Munich-Gauting, Bavaria, Germany
4. Republican Specialized Scientific Practical Medical Centre of Phthisiology and Pulmonology under Ministry of Health of the Republic of Uzbekistan, Tashkent, Republic of Uzbekistan.
5. Asklepios Lungenklinik Gauting, Pulmonary Hospital, Munich-Gauting, Bavaria, Germany
6. National Reference Laboratory on grounds of the Republican Tuberculosis Hospital Machiton, Vahdat city, Shifo, Republic of Tajikistan.

Running title

One-day TB Diagnosis and DST with MAX™ MDR-TB and AmPORE-TB

Key Words

Tuberculosis; Diagnostics; BD Max™ MDR-TB; Ampore-TB; targeted next generation sequencing; mWRD

Corresponding Author

Dr. Harald Hoffmann

Institute of Microbiology & Laboratory Medicine
IML red GmbH, SYNLAB Gauting, Robert Koch-Allee 2, 82131 Gauting, Germany

Harald.Hoffmann@synlab.com Phone: +49 89 85791 5401 Mobile; +49 160 2144428

Supplemental Material

Table S1: Characteristics of *M. tuberculosis* strains used for spiking pooled sputum aliquots and recovered from clinical samples, sample types, and smear positivity.

MTB characteristics		***Spiked samples N (%)***	***Clinical samples N (%)***
Resistance Profile		*Strains for spiking* ***52***	*Clinical Strains N (%)* ***74***
	Susceptible	*4 (7.7)*	*31 (41.9)*
	MDR	*3 (5.8)*	*6 (8.1)*
	Pre-XDR	*3 (5.8)*	*5 (6.7)*
	XDR	*4 (7.7)*	*2 (2.7)*
	Rifampicin R (EQA)	*1 (1.9%)*	*0 (0)*
	Unknown	*37 (71.2)*	*16 (21.6)*
Specimen Type		*Spiked samples*	*Clinical samples*
	Sputum	*104*	*74*
Smear Positivity	*0*	*na*	*47 (63.5)*
	<10	*na*	*2 (2.7)*
	16 AFB	*na*	*1 (1.4)*
	1+	*na*	*13 (17.6)*
	2+	*na*	*3 (4.1)*
	3+	*na*	*1 (1.4)*
	Unknown	*na*	*7 (9.5)*

na, not analyzed; EQA, external quality assurance, here, the yearly WHO SRL ring trial organized by the Institute of Tropical Medicine Antwerp

Table S2: The strain ID's and the concentrations of the bacteria (in colony-forming units per milliliter; cfu/ml) in the spiked sputum samples. The numbers reflect the absolute numbers of samples spiked with the respective strains at the respective concentrations (n=109 spiked samples). The first 104 spiked samples were tested with both BD MAX™ MDR-TB and downstream tNGS. The last five samples were excluded from tNGS due to failure to detect MTBC with BD MAX™.

Strain ID	$2.5x10^0$ cfu/mL	$2.5x10^1$ cfu/mL	$2.5x10^2$ cfu/mL	$2.5x10^3$ cfu/mL	$2.5x10^4$ cfu/mL	$2.5x10^5$ cfu/mL	Total
2066	0	1	1	1	1	0	**4**
GB 4223-0009	0	1	1	1	1	0	**4**
GB-3354-0060	1	1	2	2	2	2	**10**
IML-00031	0	0	1	0	0	0	**1**
IML-00059	0	0	0	1	0	0	**1**
IML-00095	0	0	0	0	1	0	**1**
IML-00155	0	0	0	1	0	0	**1**
IML-00180	0	4	3	3	3	0	**13**
IML-00194	0	1	0	0	0	0	**1**
IML-00195	0	0	0	0	1	0	**1**
IML-00655	0	0	0	0	1	0	**1**
IML-00755	0	0	1	0	0	0	**1**
IML-00757	0	0	0	1	0	0	**1**
IML-00758	0	0	1	0	0	0	**1**
IML-00765	0	0	0	1	0	0	**1**
IML-00767	0	0	0	1	0	0	**1**
IML-00768	0	0	0	1	0	0	**1**
IML-00771	0	2	2	2	3	0	**9**
IML-00773	0	0	1	0	0	0	**1**
IML-00777	0	1	0	0	0	0	**1**
IML-00926	0	1	0	0	0	0	**1**
IML-01063	0	0	0	1	0	0	**1**
IML-01066	0	1	0	0	0	0	**1**
IML-01074	0	0	0	0	1	0	**1**
IML-01075	0	0	1	0	0	0	**1**
IML-01082	0	0	0	1	0	0	**1**
IML-01106	0	0	0	0	1	0	**1**
IML-01123	0	1	0	0	0	0	**1**
IML-01539	0	1	0	0	0	0	**1**

IML-01705	0	0	0	0	1	0	**1**
IML-01709	0	0	0	1	0	0	**1**
IML-01710	0	0	1	0	0	0	**1**
IML-01726	0	0	1	0	0	0	**1**
IML-01735	0	0	1	0	0	0	**1**
IML-01802	0	0	0	1	0	0	**1**
IML-01904	0	1	0	0	0	0	**1**
IML-01923	0	0	1	0	0	0	**1**
IML-02002	0	0	1	0	0	0	**1**
IML-02051	0	0	0	1	0	0	**1**
IML-02052	0	0	1	0	0	0	**1**
IML-02068	0	0	0	1	0	0	**1**
IML-02256	0	0	0	1	0	0	**1**
IML-02272	0	0	0	0	1	0	**1**
IML-02276	0	0	1	0	0	0	**1**
IML-02277	0	0	1	0	0	0	**1**
IML-02285	0	0	1	0	0	0	**1**
IML-02307	0	0	0	1	0	0	**1**
IML-02329	0	0	1	0	0	0	**1**
IML-03823	0	1	1	1	1	1	**5**
IML-03833	0	1	1	1	1	1	**5**
IML-03859	1	1	1	1	1	1	**6**
IML-03865	1	1	1	1	1	0	**5**
**Total**	**3**	**20**	**28**	**27**	**21**	**5**	**104**
*IML-03823**	*1*	*0*	*0*	*0*	*0*	*0*	***1***
*IML-03833**	*1*	*0*	*0*	*0*	*0*	*0*	*1*
*GB-3354-0060**	*1*	*1*	*0*	*0*	*0*	*0*	*2*
*2066**	*1*	*0*	*0*	*0*	*0*	*0*	*1*
Total	***4***	***1***	***0***	***0***	***0***	***0***	***5***

**excluded from tNGS due to failure to detect MTBC with BD MAX™*

Table S3: *Antibiotics and the corresponding genes or gene regions targeted by the* BD MAX™ MDR-TB™ *assays and AmPORE TB (ONT) assays.*

Antibiotic Resistant Profile (ARP) name	**BD MAX™ MDR-TB *targets***	***AmPORE TB (ONT) targets***
MTBC detection	*IS6110/IS1081, defR*	*hsp65*
Spoligotyping	-	*Direct repeat locus*
Isoniazid (Inh)	*inhA, katG*	*inhA, katG, fabG1*
Rifampicin (Rif)	*rpoB*	*rpoB*
Ethambutol (Emb)	-	*embA, embB*
Pyrazinamide (Pza)	-	*pncA*
Moxifloxacin (Mfx)	-	*gyrA, gyrB*
Levofloxacin (Lfx)	-	*gyrA, gyrB*
Bedaquiline (Bdq)	-	*atpE, Rv0678*
Linezolid (Lzd)	-	*rplC*
Clofazimine (Cfz)	-	*Rv0678*
Pretomanid (Pa) & Delamanid (Dlm)	-	*ddn, fbiA, fbiB, fbiC (fbiC.1, fbiC.2) fgd1*
Streptomycin (Str)	-	*gidB, rpsL, rrs*
Amikacin (Am)	-	*rrs*
Capreomycin (Cm)	-	*gidB, tlyA, rrs*
Kanamycin (Km)	-	*eis, rrs*
Ethionamide (Eto)	-	*ethA, inhA*, *fabG1*

Table S4: Repeatability of tNGS in samples depending on the concentration of bacteria (in cfu/ml). tNGS was performed up to three times with the same sample (repeat number) by the same or different technicians (Tech). The numbers indicate the numbers of antibiotics covered by the antibiotic resistance profile produced by tNGS in the individual repetition. In smaller font: number of genes that tNGS failed to sequence, the number of wild-type genes, and the individual mutations identified. *Discrepancies are highlighted in Red and Blue.*

**Strain ID**	**Repeat number**	**Tech.**	**Number of ABs covered by tNGS & mutations identified**<br>**10^1**	**10^2**	**10^3**	**10^4**	**10^5 cfu/ml**
GB 3354-0060<br>L4; T2; DS-TB	1	1	0<br>16x fail	0<br>16x fail	2<br>14x fail	16<br>all wt	16<br>all wt
GB 3354-0060<br>L4; T2; DS	2	1	6<br>11x fail	16<br>all wt	16<br>all wt	16<br>all wt	16<br>all wt
IML-00180<br>L4, LAM-Russia; preXDR	1	1	0<br>18x fail	0<br>18x fail	14<br>2x fail<br>*katG* p.Ser315Thr<br>*rpoB* p.Ser450Leu<br>*embB* p.Gln497Arg<br>*pncA* p.Val139Ala<br>*gyrA* p.Asp94Asn<br>*rplC* p.Cys154Arg<br>*rpsL* p.Lys43Arg<br>*eis* c.-12C>T<br>*ethA* p.Trp116*	16<br>*katG* p.Ser315Thr<br>*rpoB* p.Ser450Leu<br>*embB* p.Gln497Arg<br>*pncA* p.Val139Ala<br>*gyrA* p.Asp94Asn<br>*rplC* p.Cys154Arg<br>*rpsL* p.Lys43Arg<br>*eis* c.-12C>T<br>*ethA* p.Trp116*	n/a
IML-00180<br>L4, LAM-Russia; preXDR	2	1	0<br>18x fail	0<br>18x fail	16<br>*katG* p.Ser315Thr<br>*rpoB* p.Ser450Leu<br>*embB* p.Gln497Arg<br>*pncA* p.Val139Ala<br>*gyrA* p.Asp94Asn<br>*rplC* p.Cys154Arg<br>*rpsL* p.Lys43Arg<br>*eis* c.-12C>T<br>*ethA* p.Trp116*	16<br>*katG* p.Ser315Thr<br>*rpoB* p.Ser450Leu<br>*embB* p.Gln497Arg<br>*pncA* p.Val139Ala<br>*gyrA* p.Asp94Asn<br>*rplC* p.Cys154Arg<br>*rpsL* p.Lys43Arg<br>*eis* c.-12C>T<br>*ethA* p.Trp116*	n/a
IML-00180<br>L4, LAM-Russia; preXDR	3	3	0<br>16x fail	0<br>16x fail	0<br>16x fail	16<br>*katG* p.Ser315Thr;<br>*rpoB* wt;<br>*embB* p.Gln497Arg;<br>*pncA* p.Val139Ala;<br>*gyrA* p.Asp94Asn;<br>*rplC* p.Cys154Arg;<br>*rpsL* p.Lys43Arg;<br>*eis* c.-12C>T;<br>*ethA* p.Trp116*	n/a
IML-00771<br>L4, LAM-Russia; MDR	2	3	0<br>8x fail	7<br>8x fail<br>*inhA* c.-777C>T<br>*katG* p.Ser315Thr<br>*rpoB* wt<br>*embB* fail<br>*pncA* p.Leu156fs	11<br>2x fail<br>*inhA* c.-777C>T<br>*katG* p.Ser315Thr<br>*rpoB* p.His445Leu<br>*embB* wt<br>*pncA* p.Leu156fs	16<br>*inhA* c.-777C>T<br>*katG* p.Ser315Thr<br>*rpoB* p.His445Leu<br>*embB* p.Met306Ile<br>*pncA* p.Leu156fs	n/a
IML-00771<br>L4, LAM-Russia; MDR	3	3	0<br>9x fail	7<br>9x fail<br>*inhA* c.-777C>T<br>*katG* p.Ser315Thr<br>*rpoB* fail<br>*embB* p.Met306Ile<br>*pncA* p.Leu156fs	14<br>4x fail<br>*inhA* c.-777C>T<br>*katG* p.Ser315Thr<br>*rpoB* p.His445Leu<br>*embB* p.Met306Ile<br>*pncA* p.Leu156fs	16<br>*inhA* c.-777C>T<br>*katG* p.Ser315Thr<br>*rpoB* wt<br>*embB* p.Met306Ile<br>*pncA* p.Leu156fs	nA

Tech, technician #; L4, *M. tuberculosis* lineage 4 (Euro-American); T2, t-sublineage of L4; LAM-Russia, sub-type the latin-american sublineage of L4; DS, fully drug susceptible, MDR, multi-drug resistant; pre-XDR, pre-extensively drug resistant; fail, tNGS has failed to sequence a gene; wt, wildtype; red-font: genes that tNGS called “wildtype” although it harbored a known resistance associated mutation; blue font: genes in which tNGS identified a mutation at a higher concentration but failed to sequence at the respective concentration.

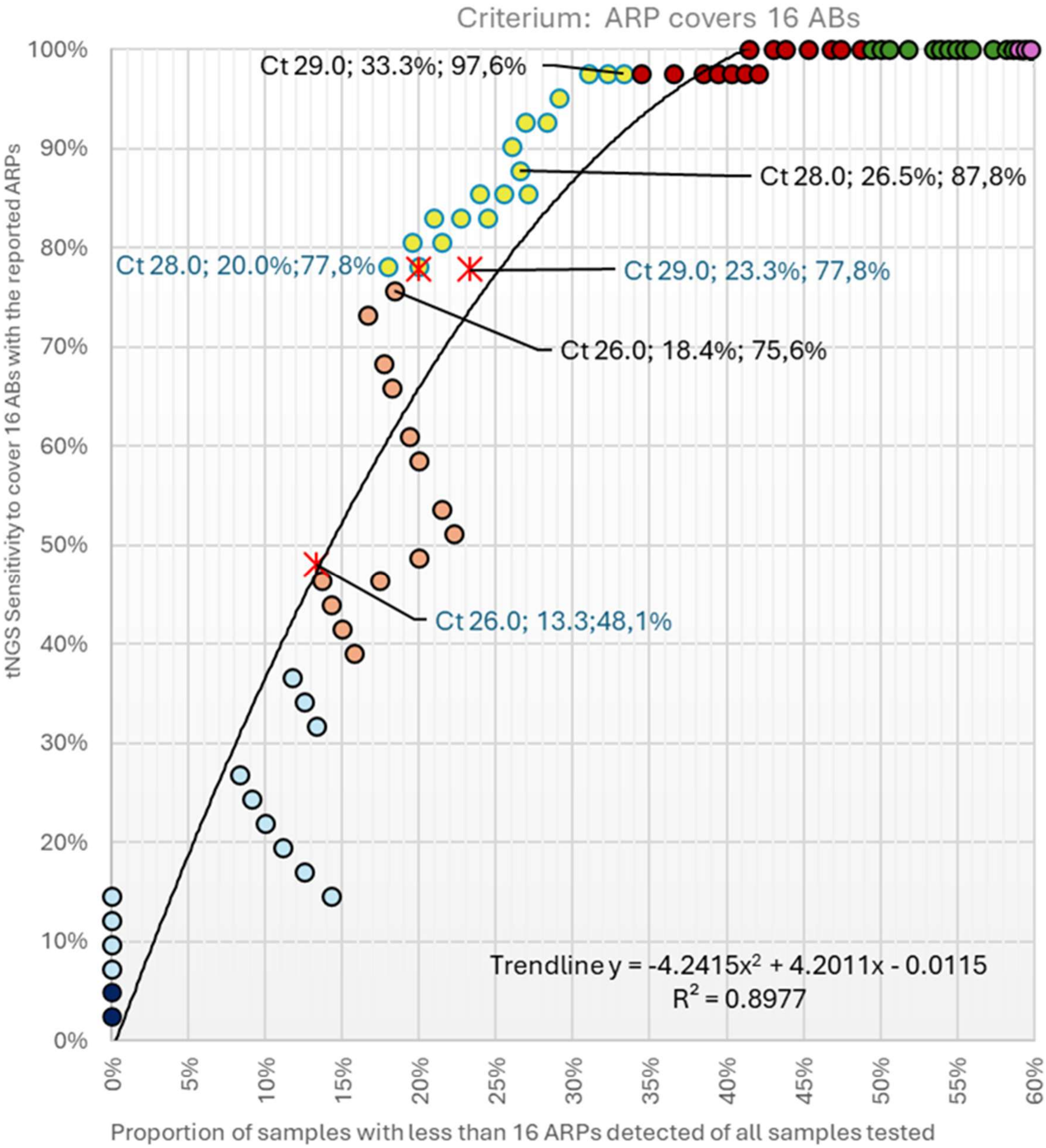

Supplemental Figure S1: BD MAX™ Ct values for the IS6110/IS1081 target and antibiotic resistance profiles (ARPs) covering 16 antibiotics (ABs). Each dot represents the Ct value of at least one spiked sample, with color indicating the corresponding Ct value range. The **y-axis** shows the proportion of samples with Ct values equal to or lower than the indicated value that yielded ARPs covering 16 antibiotics, relative to all spiked study samples with ARPs ≥12 antibiotics irrespective of their Ct-values. The **x-axis** shows the proportion of tests that failed to produce ARPs covering 16 antibiotics among all samples tested (n = 104). Red asterisks (Ж) denote the BD MAX™ Ct value thresholds of 26, 28, and 29 used for the 60 clinical samples. The percentages displayed for the highlighted dots and asterisks correspond to their respective x- and y-axis values.

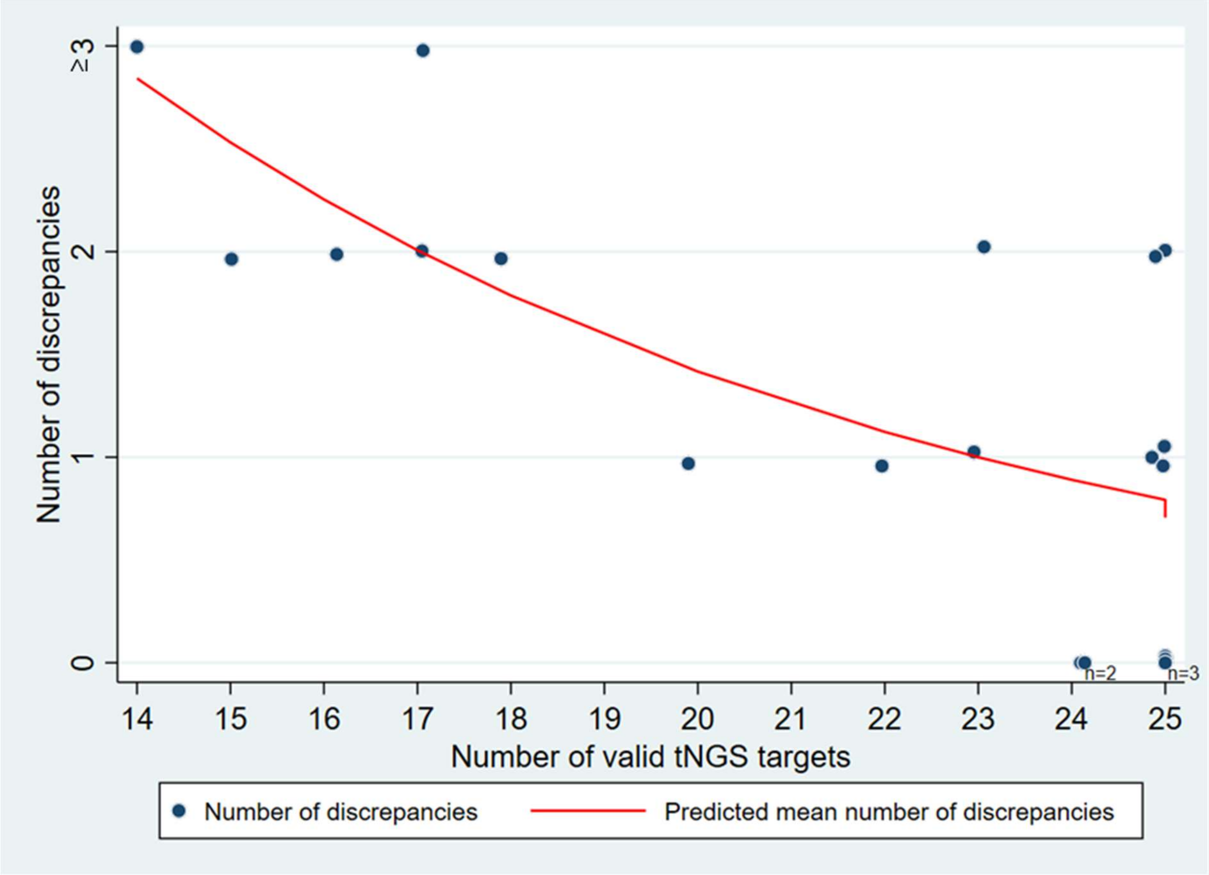

Supplemental Figure S2: Relationship between the number of valid tNGS targets and the number of discrepancies between AmPORE-TB tNGS and Illumina WGS with the PhyResSe pipelines. A Poisson regression model was used to estimate the expected number of discrepancies (red line) based on the number of valid tNGS targets (points). The model shows a significant inverse association, indicating that each additional valid target is associated with a decrease in the expected number of discrepancies ($\beta = -0.116$, $p = 0.013$; IRR = 0.89, 95% CI [0.80, 0.97]).
